# Audit of SMI Physical Health Checks and Associated Monitoring for Patients Prescribed Antipsychotics Under the Care of the Peterborough Adult Locality Team (PALT)

**DOI:** 10.1192/bjo.2026.11165

**Published:** 2026-06-30

**Authors:** Mao Fong Lim, Francesca Harris, Kazeem Ayanda, Nirmalan Ayadurai

**Affiliations:** 1 Cambridgeshire and Peterborough NHS Foundation Trust, Cambridge, United Kingdom; 2 Department of Psychiatry, University of Cambridge, Cambridge, United Kingdom

## Abstract

**Aims:**

People with severe mental illness (SMI) die on average 15–20 years earlier than the general population, largely due to poor physical health. NICE guidance mandates annual physical health checks (PHCs) for individuals with SMI, including the “Core 6” parameters (alcohol and smoking status, blood pressure, body mass index, lipid profile and HbA1c or blood glucose). NICE also requires regular physical health monitoring for those prescribed antipsychotic medication. This audit aimed to assess PALT’s compliance with NICE standards and identify barriers to completion.

**Methods:**

A retrospective clinical audit was conducted. 100 patients under the care of PALT for at least 12 months who were prescribed antipsychotic medication were included. Data was obtained from patient records between March–April 2025. Outcomes measured included completion of coded annual SMI PHCs, completion of Core 6 components, antipsychotic monitoring blood tests, and ECGs within the preceding 12 months. Engagement with PALT and evidence of invitation to SMI PHCs were also recorded. Results were analysed and compared against NICE standards and previous audits.

**Results:**

Overall, 59% of patients had an SMI PHC in the preceding 12 months, despite 98% engaging with PALT during the same period. Among those without a completed PHC, 95% had recent contact with PALT. Of the 41% who did not attend SMI PHC, only 34% had documented evidence of invitation.

Completion of individual ‘Core 6’ components was higher, ranging from 78–84%. Annual antipsychotic blood test monitoring showed good compliance at 79–84%, demonstrating improvement from previous audits. However, only 63% had an annual ECG. Even for antipsychotics with moderate-high risk of respective side effects, metabolic monitoring was suboptimal (46%), and prolactin monitoring was low (27%).

**Conclusion:**

Although compliance with antipsychotic blood monitoring is improving, completion of annual SMI PHCs remains suboptimal. Better completion of individual ‘Core 6’ components suggests that this activity is being carried out, but in a fragmented manner. The high engagement with PALT relative to SMI PHC completion suggests a missed opportunity to integrate PHC with mental health appointments. Our recommendations focus on improving awareness and accurate identification of patients requiring SMI registration and physical health monitoring as well as strengthening communication with primary care and the SMI PHC team. Additionally, building capacity within PALT to deliver physical health assessments, blood tests and ECGs, may improve engagement and completion. Continued audit and wider dissemination of findings is needed for sustained improvement in physical healthcare for people with SMI.

